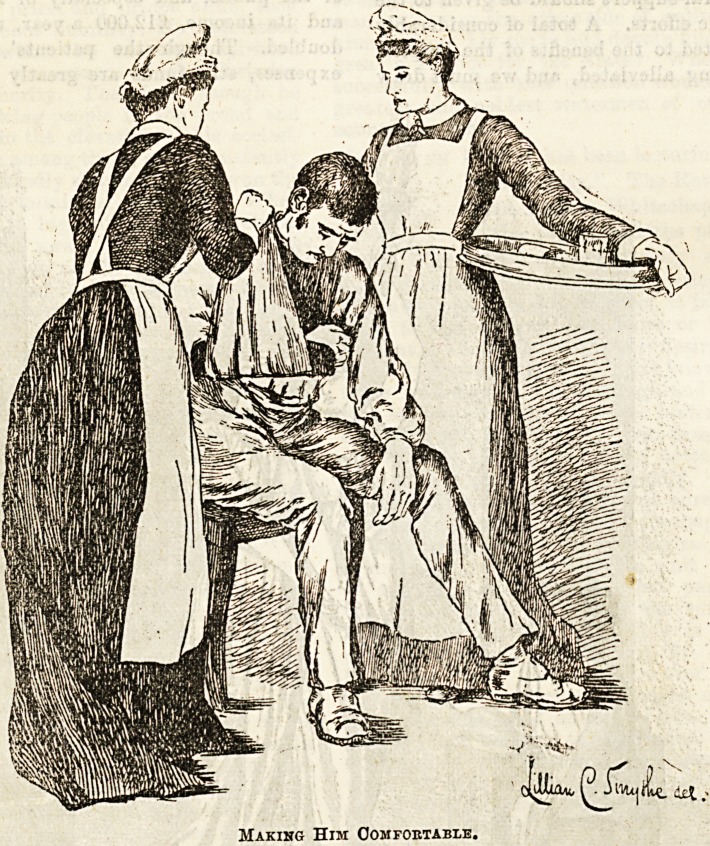# General Hospitals

**Published:** 1891-01-03

**Authors:** 


### THE PHILANTHROPIST'S VADE MECUM.

### GENERAL HOSPITALS.

**King's College Hospital,** Portugal Street, W.C.—We are indeed glad to see that this great and important hospital has been enabled to reopen those wards which the depression from which, in common with many other charitable institutions, it suffered four or five years ago reluctantly obliged it to close. That all the wards have been reopened is a cause for gratitude te all who take an interest in one of the greatest centres of medical knowledge in the Kingdom, and also a reason why liberal suppcrt should be given to the committee in their energetic efforts. A total of considerably over 21,000 patients admitted to the benefits of the hospital is a great record of suffering alleviated, and we must draw special attention to the convalescent home, which secures a longer period of ease for the patients sent to it from the hospital wards. We sincerely hope that this hospital will meet with the generous good-will it so much deserves. Secretary, Rev. N. Bromley; Matron, Miss Monk.

**Charing Cross Hospital,** Agar Street, West Strand.—This institution is situated in the centre of the most crowded thoroughfares in the metropolis, and has therefore to provide for a greater number of accidents, *i.e.*, urgent cases, than probably any other hospital of its size. All such cases are immediately admitted without delay or difficulty. About 21,000 patients are relieved every year, more than two-thirds of which are cases of accident or emergency. Can any stronger appeal be made than this to the many visitors who usually come to London, or to the public generally? We think not. Yet, the need for pecuniary help is great and pressing, and the Council earnestly appeal for donations towards meeting the deficiency between income and expenditure amounting to £6,000, and for annual subscriptions to permanently reduce it. Matron, Miss H. Gordon; Secretary, Mr. A. E. Reade.

TWIN SISTERS OF HEALING.

**Great Northern Central Hospital,** Holloway Road, N.—This hospital is entirely free to the sick poor, no letters of recommendation being necessary for admission. During last year 48,815 patients were in attendance, of which 16,424 were new cases, and 3,767 accidents. The present buildings (opened in 1888) form only a part of what the hospital will be when finished, £20,000 being required for its completion. The accommodation at the disposal of the governors is found quite insufficient, many urgent cases having to be turned away for want of room, so that it is to be hoped funds will soon be forthcoming to carry out the building of the remainder of the hospital, and thereby the doubling of the accommodation. Secretary, Mr. William T. Grant; Matron, Miss M. Hull.

**London Fever,** Liverpool Road, N., for the reception of persons suffering from infectious fevers.—It deserves well of the public, and especially of householders of all classes, and its income, £12,000 a-year, might with advantage be doubled. Though the patients' fees cover many of the expenses, still funds are greatly needed, not only to meet present wants, but to provide extra accommodation. The domestic servants of governors and employés of subscribing firms, clubs, and hotels are treated free. Secretary, Major W. Christie; Matron, Miss H. F. Ingall.

**London Hospital,** Whitechapel Road, E.—This is the greatest hospital in this country. It includes special departments under eminent medical men for the treatment of all classes of disease, the number of beds devoted to children being greater than those to be met with in most children's hospitals. The character of the work done and its value to the public may be realised from the statement that the able matron, Miss Lückes, has three assistants under her, in addition to two night superintendents, 19 day sisters, and 220 staff and probationer nurses. This great hospital of the East End is in serious want of funds, as the committee depend on voluntary contributions for £50,000 a-year to enable them to maintain the 640 beds which are daily occupied by urgent cases. Secretary, Mr. G. Q. Roberts; Matron, Miss Eva C. Lückes.

**Metropolitan Hospital,** Kingsland Road, E.—Provident principles are the foundation on which this hospital is worked, and this institution should, therefore, commend itself to the sympathies of all who believe in the provident principle as applied to medical relief. A small fixed sum paid monthly enables anyone who is ill to be seen at the hospital either in the morning or the evening. The charity is unendowed, and has no reserve fund. Secretary, Mr. Charles H. Byers.

**Royal Free Hospital,** Gray's Inn Road.—This is one of the few institutions where no governor's letter of recommendation is required, so that it is really a free hospital, the only requirements which those seeking its aid must fulfil are their being in poverty and sickness. The total expenditure of the hospital reaches £11,500 a-year, the assured income is £2,500, so that £9,000 has to be raised by constant appeals to the benevolent public. Nearly two million patients have been benefited since the Royal Free was founded, and on an average about 2,000 in and 25,000 out-patients are seen every year. Secretary, Mr. Conrad W. Thies; Matron, Miss Barton.

the class for whose benefit it is entirely working is the fact of the increasing contributions from working men. The Committee much regret that owing to there being no women's ward many urgent cases (as many as 49 women last year) had to be sent to other hospitals. During 1890 745 cases were treated as in-patients, and as many as 12,027 were attended as out-patients. Secretary, Lieut.-Colonel Edward Teneran; Matron, Miss E. Pilcher.

**St. Mary's Hospital,** Paddington.—This large general hospital, the only one of its kind in the western and north-western suburbs, labours under the great disadvantage of being situated in a bye-street of Paddington, no sentimental disadvantage, but one which results in the hospital receiving less public support than it should. The Governors are, therefore, taking steps to acquire land and premises in order to extend the institution to Praed Street. They are further stimulated to this step by the fact that the size of the hospital is most inadequate to meet the demands upon it. The amount necessary to purchase the land and erect the new wing is £85,000, and we hope the Governors will not appeal in vain to the sympathy of the public in their endeavours to fulfil, fully and efficiently, their duties to the poor of Paddington. Secretary, Mr. Thomas Ryan; Matron, Miss Medill.

Making Him Comfortable.

**Poplar Hospital for Accidents,** Blackwall, E.—This is an instance of a much-needed institution, situated in the heart of the district, which is most subject to the cases it treats. If the public only realised what daily, almost hourly, necessity there is amongst the workers in the docks and their neighbourhood for the skill and care which alone can alleviate the sufferings consequent upon their dangerous toil, abundant funds would be forthcoming for a hospital solely instituted for the relief and cure of those frightful accidents which are here of common occurrence. One evidence of the manner in which its labours are appreciated by

## Figures and Tables

**Figure f1:**
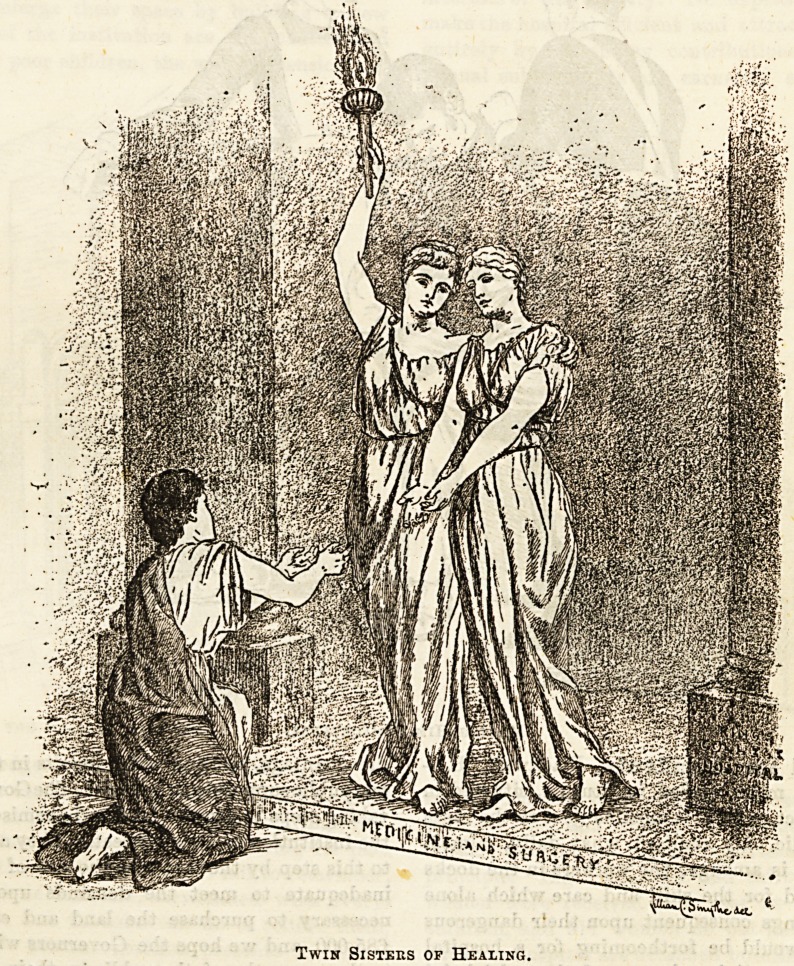


**Figure f2:**